# Targeted Routine Antenatal Anti-D Prophylaxis in the Prevention of RhD Immunisation - Outcome of a New Antenatal Screening and Prevention Program

**DOI:** 10.1371/journal.pone.0070984

**Published:** 2013-08-06

**Authors:** Eleonor Tiblad, Agneta Taune Wikman, Gunilla Ajne, Agneta Blanck, Yvonne Jansson, Anita Karlsson, Elisabeth Nordlander, Bibi Shassti Holländer, Magnus Westgren

**Affiliations:** 1 Department of Clinical Science Intervention and Technology, Division of Obstetrics and Gynecology, Karolinska Institutet, Stockholm, Sweden; 2 Department of Laboratory Medicine, Division of Clinical Immunology and Transfusion Medicine, Karolinska Institutet, Stockholm, Sweden; 3 Department of Obstetrics and Gynecology, Karolinska University Hospital, Stockholm, Sweden; 4 Department of Clinical Immunology and Tranfusion Medicine, University Hospital, Stockholm, Sweden; UCL Institute of Child Health, University College London, United Kingdom

## Abstract

**Objective:**

To estimate the incidence of RhD immunisation after implementation of first trimester non-invasive fetal *RHD* screening to select only RhD negative women carrying *RHD* positive fetuses for routine antenatal anti-D prophylaxis (RAADP).

**Materials and Methods:**

We present a population-based prospective observational cohort study with historic controls including all maternity care centres and delivery hospitals in the Stockholm region, Sweden. All RhD negative pregnant women were screened for fetal *RHD* genotype in the first trimester of pregnancy. Anti-D immunoglobulin (250–300 µg) was administered intramuscularly in gestational week 28–30 to participants with *RHD* positive fetuses. Main outcome measure was the incidence of RhD immunisation developing during or after pregnancy.

**Results:**

During the study period 9380 RhD negative women gave birth in Stockholm. Non-invasive fetal *RHD* genotyping using cell-free fetal DNA in maternal plasma was performed in 8374 pregnancies of which 5104 (61%) were *RHD* positive and 3270 (39%) *RHD* negative. In 4590 pregnancies with an *RHD* positive test the women received antenatal anti-D prophylaxis. The incidence of RhD immunisation in the study cohort was 0.26 percent (24/9380) (95% CI 0.15–0.36%) compared to 0.46 percent (86/18546) (95% CI 0.37 to 0.56%) in the reference cohort. The risk ratio (RR) for sensitisation was 0.55 (95% CI 0.35 to 0.87) and the risk reduction was statistically significant (p = 0.009). The absolute risk difference was 0.20 percent, corresponding to a number needed to treat (NNT) of 500.

**Conclusions:**

Using first trimester non-invasive antenatal screening for fetal *RHD* to target routine antenatal anti-D prophylaxis selectively to RhD negative women with *RHD* positive fetuses significantly reduces the incidence of new RhD immunisation. The risk reduction is comparable to that reported in studies evaluating the outcome of non selective RAADP to all RhD negative women. The cost-effectiveness of this targeted approach remains to be studied.

## Introduction

Alloimmunisation against the Rhesus D (RhD) red cell antigen is still the most common cause of severe hemolytic disease of the fetus and newborn (HDFN). [1], [2] Affected pregnancies require close surveillance in order to detect fetal anemia in a timely manner, and the neonates often require treatment for hyperbilirubinemia and anemia. RhD immunisation is the commonest indication for intrauterine blood transfusion and neonatal exchange transfusion. [3], [4] Once a woman is RhD immunised, all her subsequent pregnancies with an RhD positive fetus will be affected.

Since the beginning of the 1970s, the risk of RhD immunisation has been reduced from around 13 percent to 1–2 percent by administration of anti-D immunoglobulin (IgG) after delivery. [5] It has been shown that the most important residual cause of immunisation is silent fetomaternal haemorrhage (FMH) during pregnancy, mostly during the third trimester. [6], [7] Routine antenatal anti-D prophylaxis (RAADP) at the beginning of the third trimester has been introduced in several countries, reducing the incidence of RhD immunisation to 0.2–0.3 percent. There is strong evidence that RAADP is effective in the prevention of RhD immunisation. [8]–[11] Anti-D prophylaxis consists of pooled polyclonal anti-D IgG from human plasma donors. Generally, RAADP has been administered to all RhD negative women, even though 40 percent will carry a compatible RhD negative fetus, and so are not at risk for RhD immunisation. RAADP in such women unnecessarily exposes them to a human plasma product and is wasteful of the limited supply of anti-D immunoglobulin, which should be given only when strictly indicated. The discovery of cell-free fetal DNA in maternal plasma has led to the development of non-invasive methods to determine the fetal *RHD* genotype.[12]–[14] Several studies have confirmed the safety and high diagnostic accuracy of this approach.[15]–[19] This enables administration of RAADP selectively to RhD negative women with an *RHD* positive fetus and avoids giving it to women who are not at risk. This strategy has recently been introduced to Danish and Dutch screening programs, but no study has yet been published of the effectiveness of this approach in reducing the incidence of new RhD immunisation. [20].

Routine antenatal anti-D prophylaxis has not previously been offered in Sweden. The aim of this study was to estimate the incidence of RhD immunisation after implementation of first trimester non-invasive fetal *RHD* screening using cell-free fetal DNA in maternal plasma to selectively provide routine antenatal anti-D prophylaxis (RAADP) to RhD negative women carrying an *RHD* positive fetus.

## Materials and Methods

### Ethics Statement

The study was approved by the regional ethics committee in Stockholm (reference no 2009/479-31/4). All women who received antenatal anti-D prophylaxis gave written informed consent to participation in the study. All RhD negative pregnant women received written information about non-invasive fetal *RHD* genotyping and their results.

### Study Design and Participants

We performed an observational cohort study that aimed to include all RhD negative pregnant women in the region of Stockholm, Sweden, from 1st of September 2009 to 31st of December 2011. All RhD negative pregnant women without anti-D antibodies in the 1st trimester of pregnancy were eligible. The study cohort was defined as women who had received RAADP and who delivered between 1^st^ of January 2010 and 31^st^ of March 2012. The reference cohort consisted of all RhD negative women giving birth in the same region 2004–2008.

### Routine Management of RhD Negative Women in the Reference Cohort

The majority of pregnant Swedish women book their first visit to a maternity care centre early in the first trimester. Dating of the pregnancy is initially done by the last menstrual period, but later corrected according to the second trimester ultrasound. In IVF pregnancies, IVF data is used for dating. In the Stockholm region there are about 80 maternity care centres and six delivery units. The red cell alloantibody screening program during pregnancy in Stockholm has included testing in the first trimester and in RhD negative women testing around weeks 25 and 37 of gestation. No antibody test was routinely performed at delivery or post partum and no routine screening for excessive fetomaternal haemorrhage after delivery was implemented in Sweden. In nulliparous, antibody testing in the second trimester (week 25) has not been included in the routine program. All women with red cell antibodies have been referred to the Karolinska University Hospital for further management and all cases have been entered into a quality register (www.gravimm.se). Post delivery anti-D prophylaxis (250–300 µg) in non-immunised women was introduced in the early 1970s. Anti-D prophylaxis has also been administered after interventions with risk of FMH (chorionic villus biopsies, amniocentesis, cordocentesis, external cephalic version etc) as well as after surgical terminations of pregnancy and spontaneous or induced abortion after 12 weeks of gestation. The same dose of anti-D IgG was administered in single and multiple pregnancies. Cord blood serology and direct agglutination test (DAT) have been routinely performed on all newborns. The incidence of new RhD immunisations in the reference cohort was determined by searching the quality register for all RhD immunised women who seroconverted during pregnancy or after a delivery during the five-year period. No routine post partum antibody testing was performed in the reference cohort, hence immunisation after delivery was defined as presence of anti-D antibodies in the first trimester in the subsequent pregnancy. The date of the first anti-D positive plasma sample was confirmed against the transfusion database at the Department of Immunology and Transfusion Medicine. All women with anti-D antibodies after delivering abroad, due to anti-D prophylaxis or unclear cases were excluded.

### Study Protocol

Antibody testing was performed at booking in the first trimester, in the 25^th^ week of gestation independently of parity and at delivery when the women presented in the labour ward. Participants in the study were also asked to provide a plasma sample for a follow-up antibody test six to ten months post partum. After informed consent, a single injection of 250–300 µg of anti-D IgG was administered intramuscularly in pregnancy week 28–30 to RhD negative women with an *RHD* positive fetus. An extra plasma sample was taken and frozen before injection of anti-D. This sample was only analysed in women who subsequently had a positive test at delivery, to determine whether they had already been sensitised at the time of RAADP or not. The study participants also received extra anti-D prophylaxis at events during pregnancy with increased risk for FMH, and after delivery. The same dose of anti-D IgG was administered in single and multiple pregnancies. A new RhD immunisation was defined as a previously seronegative woman who developed anti-D antibodies after the 1st trimester of pregnancy or postpartum during the study period. Women with an *RHD* negative fetus did not undergo repeat antibody screenings after the first trimester and did not receive anti-D prophylaxis at events during pregnancy with risk of FMH. Women who declined to participate in the study or did not receive RAADP for other reasons such as moving to the region late in pregnancy or who had received anti-D prophylaxis abroad received routine care.

### Non-invasive Fetal RHD Genotyping

From 1^st^ of September 2009 genotyping on cell-free fetal DNA in maternal plasma was performed in the first trimester of pregnancy. Two 4.5 mL EDTA-anticoagulated blood samples were obtained as part of routine blood sampling at the booking visit. One sample was used for routine ABO RhD blood typing and red blood cell antibody screening and the other was used for fetal *RHD* determination in women who typed RhD negative. Blood typing and antibody screening were done according to established routines on an automated blood group typing instrument (AutoVue, Ortho Clinical Diagnostics, Raritan, NJ, USA). If red blood cell antibody screening was positive, antibody identification was performed using indirect antiglobulin technique on the AutoVue instrument or a manual gel technique (Bio-Rad Laboratories, Herts, UK) with in-house panels. The blood samples for fetal *RHD* determination were transported to the central laboratory, centrifuged, and the plasma stored at –30°C until DNA extraction. DNA extraction and PCR assay using a single-exon 4 strategy were performed as previously described. [21] From gestational week eight, the sensitivity and specificity of the fetal *RHD* genotyping was 98.9 percent respectively. Analyses were done two or three times a week and fetal *RHD* type reported to the maternal care units within one to two weeks. Results were normally available in gestational weeks 10–13.

### Outcome Measures and Data Collection

The main outcome measure was the incidence of RhD immunisation developing during or after pregnancy in both cohorts. All pregnancies with an *RHD* positive genotype were entered into a study database and follow-up data were recorded prospectively. Results from antibody screening, cord blood serology and the direct agglutination test were retrieved from the transfusion medicine database. Data on obstetric and neonatal outcome were retrieved from electronic medical records. Statistics on RhD negative deliveries during the study period and reference period were retrieved from the regional delivery database (Obstetrix) and from the Swedish Medical Birth Register. The prevalence of RhD negative pregnant women in Sweden has been stable at 14.6 percent [22].

### Study Size

The birth rate in Stockholm have varied between 25–27 000 births per year, representing approximately 25 percent of all births in Sweden. A power analysis was performed before the start of the study to determine the necessary study size. We hypothesised a reduction of RhD immunisation from 1 to 0.5 percent. [22] Using a two-sided test for binomial distribution at a significance level of 0.05 and 80 percent power, it was estimated that 4540 RhD negative women would be required in each cohort.

### Statistical Analyses

An intention-to-treat analysis was applied to the estimation of reduction of incidence of immunisations. All cases of new RhD immunisations during the study period were included in the analysis, even if the woman had not received RAADP. Frequencies and absolute numbers of missing data are reported in tables and figures. Incidence was calculated by dividing all new cases in each period by the total number of RhD negative pregnant giving birth during each respective period. Only individuals who received RAADP and hence had given their informed consent are included in the descriptive statistics. Statistica 10.0 was used for the statistical analysis (StatSoft®, Inc. Tulsa OK, USA). The analyses comprised descriptive statistics and a Chi-square test to compare the incidence in the two groups. A p-value of 0.05 was used to define statistical significance.

## Results

**Figure 1:**
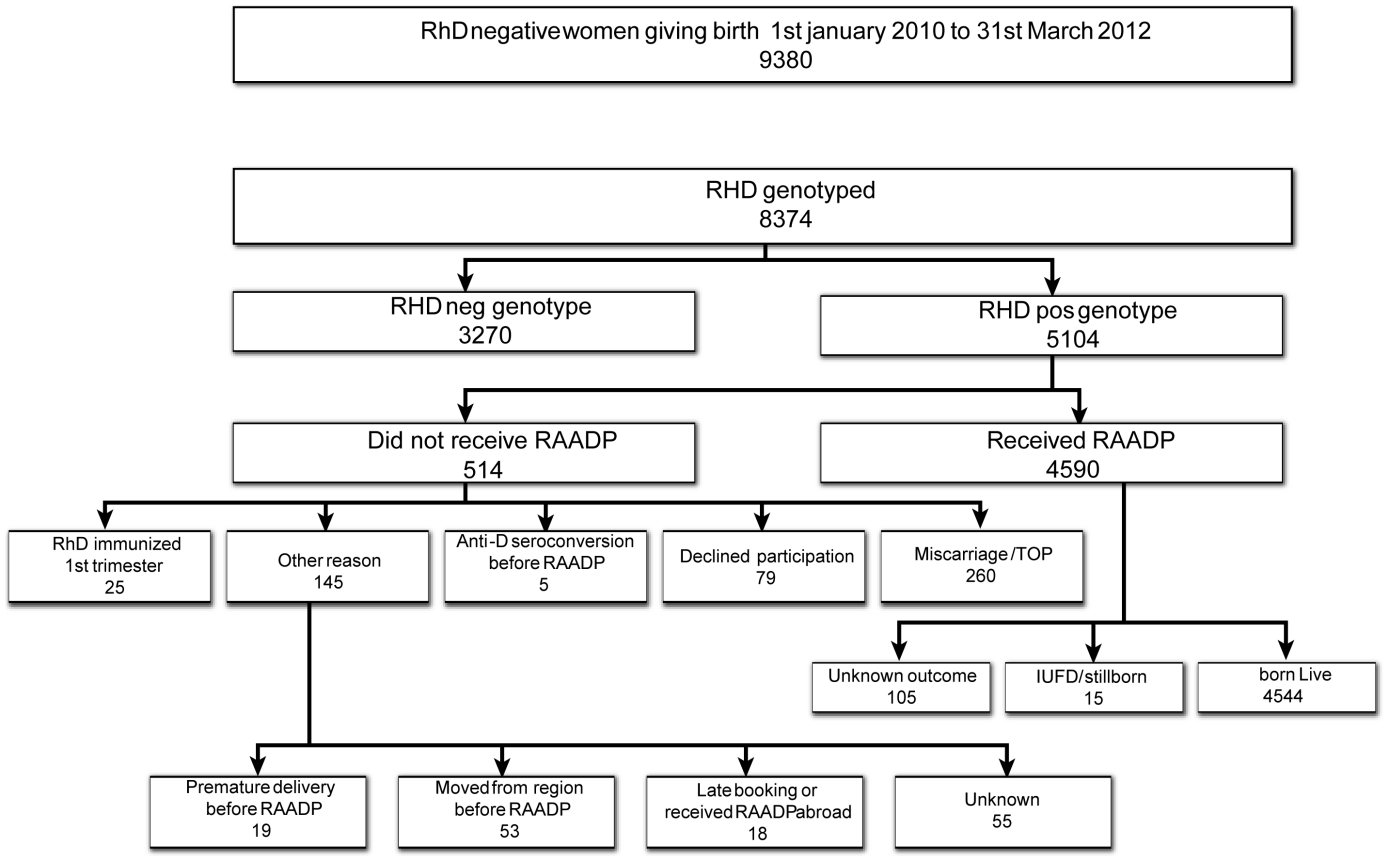
Study participants and outcomes.

Between 1^st^ of January 2010 and 31^st^ of March 2012, 9380 RhD negative women gave birth in Stockholm. Non-invasive fetal *RHD* genotyping was performed in 8374 pregnancies (89%) (1 Sept 2009 to 1 Sept 2011). 3270 fetuses were determined to be *RHD* negative (39%) and 5104 (61%) *RHD* positive. 4521 women participating with 4590 pregnancies (90%) received targeted RAADP due to an *RHD* positive fetus. Sixty-nine women participated with two pregnancies. Twenty-five women with anti-D antibodies in the first trimester of pregnancy and *RHD* positive fetal genotype were excluded from the study. Data on all included and excluded women and outcomes are described in Figure 1. The median gestational week for receiving RAADP was 29 weeks (25^th^ to 75^th^ percentile: 29 to 30 weeks).

In the reference cohort, the incidence of RhD immunisation was 0.46 percent (95% CI 0.37 to 0.56) (86/18546). Twenty-four new cases of RhD immunisation have been confirmed during the study period, resulting in an incidence of 0.26 percent (24/9380) (95% CI 0.15–0.36%). Of these new cases, two women had not received RAADP and fetal *RHD* genotyping had not been performed. This happened at the beginning of the study period when compliance with the new program was incomplete. Frequencies of antibody screening tests in the study cohort were 88 percent in the second trimester (around 25 weeks of GA), 74 percent at delivery, 71 percent post partum (median 7.5 months after delivery). In 91 percent of participants at least one sample was taken at delivery or at post partum follow-up. The frequency of antibody tests in the second and third trimester in the reference cohort was unknown. The risk ratio (RR) for sensitisation with the new program was 0.55 (95% CI 0.35 to 0.87). The absolute risk difference was 0.20 percent, which corresponds to a number needed to treat (NNT) of 500. The risk reduction was statistically significant (P = 0.009).

Data on all new RhD immunisations during the study period are described in Table 1. Fifty-eight percent (14/24) of the women were already sensitised before being scheduled for anti-D prophylaxis. Four women were sensitised in spite of receiving RAADP and had seroconverted at delivery. In two of these, the frozen sample was not available and the possibility of sensitisation before prophylaxis cannot be ruled out. Two women had a negative antibody test at delivery but anti-D was detected at 10 months postpartum or in the first trimester in a subsequent pregnancy. In two cases it was unclear if the women had become immunised during the third trimester in spite of receiving RAADP or after delivery. The antibody test at delivery was missing in these two cases. In the two cases where the women had not received RAADP, one woman already had detectable anti-D antibodies at 28 weeks of gestation. Seven of the immunised women were nulliparous. One of those had anti-D antibodies with high titres detected at routine screening at 25 weeks of gestation. She had rapidly increasing antibody concentrations and multiple intrauterine blood transfusions were required due to fetal anemia. A boy was delivered at 34 weeks and congenital cytomegalovirus (CMV) infection was diagnosed soon after birth in addition to severe hemolytic disease of the newborn. Investigation of the mother, neonate and the blood donors indicated reactivated CMV infection in the mother during pregnancy.

**Table 1:** Details on women RhD immunised during study period.

No	Gravida	Para	GA* RAADP#	1st pos antibody test GA	GA delivery	Comment
**1**	3	1	29	1st trimester subsequent pregnancy	38+6	Immunised after delivery. Neg screen at delivery.
**2**	6	1	31	At delivery	38+6	Immunised 3rd trimester. Frozen sample not available.Neg screen at 25 weeks GA.
**3**	1	0	30	30	41+0	Immunised before RAADP, Frozen sample positive.
**4**	2	1	29	29	40+4	Immunised before RAADP. Frozen sample positive.
**5**	4	2	28	10 months postpartum	39+0	Immunised after delivery. Neg screen at delivery.
**6**	7	2	Not received	26	36+0	Immunised before RAADP
**7**	2	1	Not received	25	37+0	Immunised before RAADP
**8**	1	0	Not received	24	36+4	Immunised before RAADP
**9**	1	0	31	At delivery	42+2	Immunised 3rd trimester. Frozen sample not available.Neg screen at 26 weeks GA.
**10**	1	0	29	1st trimester subsequent pregnancy	39+2	Unclear if immunised during third trimester or after delivery. Missing screen at delivery. Frozen sample negative.
**11**	2	1	29	29	39+6	Immunised before RAADP. Frozen sample positive.
**12**	3	2	Not received	24	Unknown	Immunised before RAADP. Moved from region.
**13**	2	0	32	32	37+2	Immunised before RAADP. Frozen sample positive.
**14**	3	1	30	30	41+5	Immunised before RAADP. Frozen sample positive.
**15**	2	1	29	At delivery	41+1	Immunised 3rd trimester. Frozen sample negative.
**16**	5	3	29	25	37+0	Immunised before RAADP
**17**	1	0	Not received	25	34+0	Immunised before RAADP. Severe HFDN¶ requiring multiple intrauterine transfusions. Neonate with congenital cytomegalovirus infection.
**18**	2	1	29	29	40+2	Immunised before RAADP, Frozen sample positive.
**19**	3	2	29	29	38+4	Immunised before RAADP, Frozen sample positive.
**20**	2	0	30	30	39+0	Immunised before RAADP, Frozen sample positive.
**21**	5	2	29	At delivery	40+2	Immunised 3rd trimester. Frozen sample negative.
**22**	4	2	29	7 months postpartum	41+5	Unclear if immunised during third trimester or after delivery. Missing screen at delivery. Frozen sample negative.
**23**	2	1	Not received	28	38+0	Fetal *RHD* genotyping not performed. Missed at beginning of study.
**24**	2	1	Not received	36	37+5	Fetal *RHD* genotyping not performed. Missed at beginning of study.

* GA = gestational age.

# RAADP = routine antenatal anti-D prophylaxis.

¶ HDFN = haemolytic disease of the fetus and newborn.

## Discussion

First trimester non-invasive antenatal screening for fetal *RHD* combined with routine antenatal anti-D prophylaxis given selectively to women with an *RHD* positive fetus significantly reduces the incidence of RhD immunisations. The risk reduction is comparable to that reported in studies evaluating the outcome of giving RAADP to all RhD negative women.[9]–[11], [23] To our knowledge, this is the first large study to evaluate the reduction of RhD immunisation in a community after introduction of such a program. The number needed to treat (NNT) to prevent one sensitisation was 500, which means that non-invasive *RHD* screening would have to be performed in 500 women, but only about 60 percent (NNT 300) of these would need RAADP.

Of the two cases in which the women had not received antenatal prophylaxis, one might have been avoided with prophylaxis. In the third woman, anti-D was already detectable at 28 weeks of gestation and sensitisation would therefore not have been prevented with the current program. Large fetomaternal haemorrhages that overwhelm the dose of anti-D IgG administered may lead to sensitisation in spite of prophylaxis. Analysis for fetal red blood cells in maternal blood after delivery to assess the magnitude of FMH is not routinely performed in Sweden, but in a few women the standard dose of 250–300 µg will not be sufficiently protective. Risk factors for RhD immunization in spite of prophylaxis include assisted delivery and caesarean section, postmaturity, pregnancy-related red blood cell transfusion and younger age. [24] Three women immunized in spite of RAADP in our study cohort delivered after 41 weeks of gestation. The effect of RAADP depends not only on the size of silent FMH during the third trimester, but also on the time between administration and delivery. Most women have detectable levels of anti-D IgG 10 weeks after receiving 250–300 µg RAADP but after that levels are variable. [25] [26] This means that some pregnant women will be unprotected in gestational weeks 38 to 42. Routine administration of anti-D prophylaxis in gestational weeks 38–39 instead of the postnatal injection could protect throughout this period. A high enough dose at this gestational age will normally protect against sensitisation after delivery.

Screening in the first trimester has the clinical advantage that only individuals at risk are exposed to a human plasma product. Women will also benefit from knowing the *RHD* type of the fetus in case of miscarriages, abortions and invasive prenatal testing such as amniocentesis and chorionic villus sampling. A disadvantage of first trimester screening is the lower sensitivity of the test compared to later in pregnancy. [21] However, for a screening purpose, the sensitivity of 98.9 percent is still very high and first trimester testing is safe to use. For a diagnostic purpose, as in already RhD immunised pregnant women, we recommend repeated testing in gestational week 20 for confirmation when fetal *RHD* genotype is determined as negative. Knowing the fetal genotype early in pregnancy also enables new preventive strategies to reduce sensitisation in the second trimester.

A relatively high number (14/9380, incidence approximately 0.15%)) of cases of new RhD immunisations in the study cohort occurred during the second trimester of pregnancy. Our findings are in line with the evaluation of the Dutch RhD prevention program, in which the incidence of immunisations occurring between 12 to 30 weeks of pregnancy was 0.24 percent before introduction of RAADP and 0.25 percent after. [10] In Bowman’s classical studies, a comparable frequency of women were immunised before the 28^th^ week of gestation. The most likely reason for early sensitisation in spite of a negative first trimester antibody test is FMH occurring early in pregnancy. But it is also possible that some women may have been sensitised in a previous pregnancy but did not develop detectable IgG antibodies until the second trimester of the subsequent pregnancy. Routine anti-D prophylaxis could be added in 16 weeks of pregnancy to prevent second trimester sensitisation in women with *RHD* positive fetuses to protect until the dose at week 28.

We performed a community-based study aiming at evaluating the outcome of a new prevention program in a routine setting. The aim was to include all RhD negative women in our population, but during the first six months of the study period recruitment to the program was slow, which explains the discrepancy between the number of RhD negative women who gave birth during the study period and the number of women who underwent screening for fetal genotype. The compliance of both women and health care professionals with the protocol has increased with time. The use of an intention-to-treat analysis should mean our study provides a good indication of the expected effectiveness of this new program in our health care system. An evaluation of the first six months of the Danish national antenatal *RHD* screening program noted that adoption of the program was slow with only about 80 percent of the expected number of maternal samples being sent to the laboratories. Compliance with antenatal administration of anti-D was unknown and can be anticipated to be lower. [20] The strengths of our study include a large study sample, prospective collection of data, central management of all cases of red cell immunisation in our region and reliable registers of these pregnancies. We used two time points, both at delivery and six to ten months postpartum, to find women who seroconverted in the study cohort. The frequency of received samples at follow-up post partum was 71 percent and at least at delivery or postpartum 91 percent. In the reference cohort, antibody screening was performed only at 37 weeks of pregnancy and not at delivery or post partum. Not all women in the reference cohort had a subsequent pregnancy when antibodies from sensitisation late in the third trimester or at delivery in the previous pregnancy would be found. We believe the detection rate of new RhD immunisations in the reference cohort to be somewhat underestimated due to this. Since the optimal time point to assess the incidence of alloimmunisation is in the first trimester of a subsequent pregnancy, we expect a few more cases to appear in the study cohort on long-term follow-up since some women may have been sensitised at delivery, but anti-D antibodies will not be detected until the first trimester of a subsequent pregnancy. Some new RhD immunisations may also be detected in future in women in the reference cohort who have another child. The incidence of RhD immunisation was as low as 0.46 percent in the reference cohort. The prevalence of RhD immunisation during the five-year period 2004–2008 was 0.7 percent. Since the study sample was large enough this did not affect the power of the study. The low frequencies compared to previous reports might be due to improved obstetric management of RhD negative women in situations with risk for FMH and also improved compliance to anti-D prophylaxis recommendations over the years. Most reports on the prevalence of RhD immunisation with a postnatal prophylaxis program were published in the 1970s and 1980s and reports already in the 1990s show a lower prevalence compared to the earlier studies. [27], [28] The incidence of RhD immunisation in the Dutch population before introduction of routine antenatal anti-D prophylaxis in 1998 was reported to be 0.5 percent, which is similar to our findings. [10].

We chose an observational cohort study to assess the effectiveness of the new intervention and not a randomised controlled study. In our opinion, there is already strong evidence for the effectiveness of RAADP in preventing sensitisation to the RhD antigen.[8]–[11], [23] A randomised study in which RAADP was administered to all RhD negative women in one arm, and targeted RAADP offered exclusively to women with an *RHD* incompatible fetus in the other would have been complicated to carry out in our setting and it is questionable whether such a study would have been successful or have provided more reliable outcome data. The use of a historical control group has its limitations. However, obstetric and transfusion practice and laboratory methods have remained essentially unchanged during the reference and study periods. We believe no important selection bias was introduced in our study.

### Conclusions

We conclude that non-invasive antenatal screening for fetal *RHD* combined with selective routine antenatal anti-D prophylaxis in the beginning of the third trimester to women with *RHD* positive fetuses significantly reduces the incidence of new RhD immunisation. The use of the targeted approach means that women not at risk for sensitisation avoid unnecessary exposure to anti-D immunoglobulin and health care resources may be more wisely used. The long-term results of the program in terms of the reduction in the incidence of severe hemolytic disease of the newborn with its attendant need for intrauterine blood transfusion and intensive neonatal care will be evaluated in the future. An economic evaluation that compares offering all RhD negative women RAADP and targeting RAADP remains to be done.
